# Impact of COVID-19 on the HIV care continuum in Asia: Insights from people living with HIV, key populations, and HIV healthcare providers

**DOI:** 10.1371/journal.pone.0270831

**Published:** 2022-07-20

**Authors:** Chien-Ching Hung, Sumita Banerjee, Ishwar Gilada, Kimberly Green, Yoji Inoue, Adeeba Kamarulzaman, Kate Leyritana, Nittaya Phanuphak, Timothy Wong, TinHung Wong, Shikha Singh, Jun Yong Choi

**Affiliations:** 1 Department of Internal Medicine, National Taiwan University Hospital and National Taiwan University College of Medicine, Taipei, Taiwan; 2 Department of Tropical Medicine and Parasitology, National Taiwan University College of Medicine, Taipei, Taiwan; 3 Action for AIDS, Singapore, Singapore; 4 AIDS Society of India, Mumbai, India; 5 PATH, Vietnam; 6 Juntendo University, Tokyo, Japan; 7 Centre of Excellence for Research in AIDS, Faculty of Medicine, University of Malaya, Kuala Lumpur, Malaysia; 8 Sustained Health Initiatives of the Philippines, SHIP, Metro Manila, Philippines; 9 Institute of HIV Research and Innovation, Bangkok, Thailand; 10 Hong Kong AIDS Foundation, Hong Kong SAR, China; 11 Gilead Sciences, Hong Kong SAR, China; 12 Cerner Enviza, Singapore, Singapore; 13 Department of Internal Medicine and AIDS Research Institute, Yonsei University College of Medicine, Seoul, Republic of Korea; Shaheed Montarma Benazir Bhutto Medical University, PAKISTAN

## Abstract

**Background:**

The COVID-19 pandemic has threatened continued access to public health services worldwide, including HIV prevention and care. This study aimed to evaluate the impact of the COVID-19 pandemic on HIV service access and delivery in the Asia region.

**Methods:**

A descriptive, cross-sectional, online study, conducted between October-November 2020, assessed the impact of COVID-19 on HIV prevention and care among people living with HIV (PLHIV), key populations (KPs), and healthcare providers (HCPs). The study populations were recruited across ten Asian countries/territories, covering Hong Kong, India, Japan, Malaysia, Philippines, Singapore, Korea, Taiwan, Thailand, and Vietnam.

**Results:**

Across the region, 702 PLHIV, 551 KPs, and 145 HCPs were recruited. Both PLHIV and KPs reported decreased or had yet to visit hospitals/clinics (PLHIV: 35.9%; KPs: 57.5%), reduced HIV RNA viral load testing (21.9%; 47.3%), and interruptions in antiretroviral therapy (ART) (22.3%) or decreased/complete stop of HIV prevention medication consumption (40.9%). Travel constraints (40.6%), financial issues (28.9%), and not receiving prescription refills (26.9%) were common reasons for interrupted ART access, whereas reduced engagements in behaviours that could increase the risks of HIV acquisition and transmission (57.7%), travel constraints (41.8%), and less hospital/clinic visits (36.7%) underlie the disruptions in HIV preventive medications. Decreased visits from PLHIV/KPs and rescheduled appointments due to clinic closure were respectively reported by 50.7%-52.1% and 15.6%-17.0% of HCPs; 43.6%-61.9% observed decreased ART/preventive medication refills. Although 85.0% of HCPs adopted telemedicine to deliver HIV care services, 56.4%-64.1% of PLHIV/KPs were not using telehealth services.

**Conclusions:**

The COVID-19 pandemic substantially disrupted HIV prevention to care continuum in Asia at the time of the study. The findings highlighted differences in HIV prevention to care continuum via telehealth services utilisation by PLHIV, KPs, and HCPs. Efforts are needed to optimise infrastructure and adapt systems for continued HIV care with minimal disruptions during health emergency crises.

## Introduction

The outbreak of the Coronavirus Disease 2019 (COVID-19) pandemic not only directly caused high morbidity and mortality, but also threatened general public health services and care delivery globally [1]. The severity of the pandemic resulted in overburdened healthcare systems and imposed “lockdown” measures to limit the spread of COVID-19 within the community or region leading to reduced access to healthcare [2, 3].

HIV prevention and care is key towards eliminating the public health threat of HIV and comprises of measures including testing; HIV prevention with medications including pre-exposure prophylaxis (PrEP) or non-occupational post-exposure prophylaxis (nPEP); diagnosis; and treatment using antiretroviral therapy (ART) and achieve viral suppression [4, 5]. Universal access to HIV care and treatment is essential for people living with HIV (PLHIV) to effectively manage HIV infection and drastically reduce HIV-related morbidity and mortality [6].

The imposed lockdown measures have impacted the manufacturing and supply chain of antiretrovirals and their distribution, resulting in significant delays in the delivery of medicines and related health services [7]. For example, the PrEP roll-out to eliminate HIV transmission during the pandemic was ‘indefinitely postponed’ in the UK [8], and 35.1% of people living with HIV (PLHIV) in China experienced or were at risk of ART interruptions due to travel restrictions, insufficient ART reserve, and/or suspension of courier services [9].

Across the world, HIV infrastructure was repurposed or leveraged to manage and sustain COVID-19 responses, disrupting the continuity of HIV care [7, 10, 11]. Most HIV physicians who were also infectious diseases physicians in Central and Eastern Europe reportedly undertook dual care duties for HIV as well as COVID-19 [10], and medical facilities in China were designated as COVID-19 response centres and had suspended taking on new patients with HIV and other infectious diseases [12].

A modelling study in 2020 forecasted an additional 10% HIV-related deaths over a 5-year timeframe in the event of a disruption in HIV treatment than those without disruptions [13]. Similarly, a six-month disruption in programs to prevent mother-to-child transmission of HIV would cause new infections in children to increase by 40%-80% in high-burden countries [14]. Many studies reported a negative impact of COVID-19 pandemic on HIV care access and delivery in Africa, China, Europe and the US [12, 14–16], highlighting disruptions experienced mainly by PLHIV. However, similar studies exploring the impact of COVID-19 on the HIV prevention to care continuum in Asia are limited.

Herein, the study aimed to assess and identify gaps or barriers faced by PLHIV, key populations (KPs), and healthcare providers (HCPs) with respect to access and delivery of HIV prevention and care due to the COVID-19 pandemic in Asia. The findings from this study sought to provide insights to facilitate the narrowing of gaps identified for HIV prevention and care delivery among PLHIV, KPs (e.g., people who inject drugs, sex workers, men who have sex with men, and transgender people) and HCPs.

## Methods

### Study design

This is a cross-sectional, multi-national study comprising of an online, cross-sectional 10-minute survey, which was conducted between October-November 2020. The study was administered centrally to PLHIV, KPs, and HCPs across 10 countries/territories in Asia–Hong Kong, India, Japan, Malaysia, Philippines, Singapore, South Korea (hereafter Korea), Taiwan, Thailand, and Vietnam.

This multi-national, online survey-based study was completely voluntary and non-interventional. We have carefully examined the local and national legislations, in particular data protection in each country/territory and used informed consent as legal basis for data collection. The protocol and questionnaire of this cross-sectional, regional, online survey-based study (**#20-KANT-238)** were submitted to Pearl Institutional Review Board (IRB) (Indianapolis, Indiana) for exemption determination in accordance with FDA 21 CFR 56.104 and 45CFR46.104(b)(2): (2) Tests, Surveys, Interviews. The study was determined to be Exempt for the periods the data is used in the current study. All respondents who participated in the study provided online informed consent and only deidentified data was collected and analysed. Respondents’ rights to refuse and withdraw from the study at any time were accepted. Study participants or the public were not involved in the design, or conduct, or reporting, or dissemination plans of our research.

### Study populations

Eligible PLHIV and KP respondents (aged 21 years or older) and HCPs, who had provided informed consent, were recruited through patient advocacy groups (PAGs), HIV care centres, community clinics or medical societies. Respondents were invited to participate in the study either through email/online chat apps from PAGs/HIV care centres/community clinics/medical societies or on websites of PAGs across the 10 countries/territories.

The respective respondent groups are defined as follows:

- PLHIV: individuals who have been exposed to activities that increased the risk of transmission or acquisition of HIV and tested positive for HIV during routine testing.
- KPs: individuals who have been exposed to activities that increased the risk of transmission or acquisition of HIV, but tested negative for HIV during routine testing, e.g., people who inject drugs, sex workers, men who have sex with men, and transgender people.
- HCPs: physicians who have had clinical experience or direct clinical care with PLHIV and/or individuals who are at-risk of HIV exposure or infection (KPs).

### Survey questionnaire

The questionnaire comprised of items assessing the impact of COVID-19 on HIV care access and delivery. The questionnaire was developed in English and translated into local language(s) by linguists who are native speakers to the language(s). To ensure consistency of the questionnaire, the developed questionnaires (in English and local language translations) were reviewed and finalised by the steering committee comprising of HIV/infectious diseases experts from the 10 countries/territories and local HIV/AIDS patient advocacy organizations.

The questionnaires for PLHIV and KPs were translated into local language(s) for Hong Kong (Traditional Chinese), India (Hindi), Japan (Japanese), Malaysia (Malay, Simplified Chinese), Philippines (Tagalog), Singapore (Simplified Chinese), Korea (Korean), Taiwan (Traditional Chinese), Thailand (Thai), and Vietnam (Vietnamese) for PLHIV and KPs. For HCPs, the survey was translated into local language for Japan (Japanese), Korea (Korean), Taiwan (Traditional Chinese), and Vietnam (Vietnamese). The survey was administered in either English or the local language(s).

The questionnaire was sectioned into three main sections with specific focus on how COVID-19 impacted the respective study populations:

- Care access among PLHIV:
  - Frequency/consultation time per visit, and interval of visits to the hospital/clinic
  - Access to ART
  - Reasons for changes (if any) in visitation frequency to hospital/clinic and/or access to ART
  - Use of telehealth services (i.e., appointment, refill of ART, where applicable), with HCPs
- Preventive care access among KPs:
  - Ability/willingness to get tested for HIV
  - Ability/willingness to obtain preventive care, including prevention medications (e.g., PrEP/nPEP)
  - Reasons for changes (if any) in access to testing or preventive care
  - Use of telehealth services (where applicable) with HCPs
- Care delivery by HCPs:
  - Number of PLHIV or KPs seen (patient load), and/or frequency of consultation or consultation time per visit
  - Patient’s access to HIV testing and laboratory tests
  - Prescription of ART for PLHIV or preventive medications for KPs
  - Telehealth services adopted for HIV prevention to care delivery and perceived relevance for future HIV prevention and care.

### Descriptive analyses

Descriptive data analyses of the respondents’ characteristics and responses to the survey questions were summarized and presented as text, tables, and charts in counts and row percentages. Given the sample size of each study population in each country/territory was different, a weighting factor was applied to the responses of the respondents to treat the findings to be equally representative in the region.

Respondents’ characteristics of PLHIV and KPs included age (mean ± standard deviation [SD], 21–30 years, 31–40 years, 41–50 years, 51–60 years, ≥61 years), gender (male, female, transman, transwoman, or gender-nonconforming) and sexual orientation (bisexual, gay, lesbian, straight, or others e.g., pansexual, nonsexual, etc). Characteristics of HCPs included clinical specialty (infectious disease/HIV specialist or general practitioner), location of clinical practice i.e., type of hospital/clinic (public, government, or restructured hospital, private hospital, private clinic, medical centre, regional hospital, or area hospital), and years of clinical practice (mean ± SD, ≤5 years, 6–10 years, 11–15 years, 16–20 years, 21–25 years, >25 years) (Table 1).

**Table 1:** Characteristics of people living with HIV (PLHIV), individuals from key populations (KPs), and healthcare providers (HCPs) in Asia region (regional weighted average^†^).

		PLHIV	KPs
		N = 702	N = 551
**Age (years)**	*Mean ± SD*	* 37*.*7 ± 10*.*3*	*33*.*9 ± 8*.*9*
	*21–30*	28.0%	42.9%
	*31–40*	36.7%	36.1%
	*41–50*	22.6%	16.3%
	*51–60*	9.1%	3.4%
	*≥61*	3.7%	1.4%
**Gender**	*Male*	89.1%	77.2%
	*Female*	6.0%	6.2%
	*Transman*	0.2%	2.3%
	*Transwoman*	2.6%	10.2%
	*Gender-nonconforming*	1.3%	1.5%
	*Prefer not to answer*	0.7%	2.6%
**Sexual Orientation**	*Bisexual*	15.0%	16.8%
	*Gay*	76.9%	67.0%
	*Lesbian*	0.3%	0.4%
	*Heterosexual*	5.9%	9.6%
	*Other (e*.*g*., *pansexual*, *nonsexual*, *or trans-sexual)*	1.0%	4.1%
	*Prefer not to answer*	0.9%	2.1%
** **	* *	**HCPs**	
** **	* *	N = 145	
**Specialty**	*Infectious Disease/HIV Specialist*	90.0%	
	*General Practitioner*	10.0%	
**Type of hospital/clinic** ^**§**^	*Public/ Government / Restructured hospital*	43.1%^§^	
	*Private hospital*	28.3%^§^	
	*Private clinic*	18.0%^§^	
	*Medical centre*	65.6%^‡^	
	*Regional hospital*	28.1%^‡^	
	*Area hospital*	6.3%^‡^	
	*Other*	10.6%^§^	
**Years of Practice**	*Mean ± SD*	12.8 ± 7.6	
	*≤ 5 years*	20.6%	
	*6–10 years*	20.9%	
	*11–15 years*	29.5%	
	*16–20 years*	14.0%	
	*21–25 years*	8.1%	
	*> 25 years*	6.8%	

^†^Weighted average across all 10 countries/territories (n = 145)

^§^Weighted average (excluding Taiwan) (n = 113)

^‡^Average based on hospital/clinics applicable to Taiwan (n = 32)

SD, standard deviation

Data missing was at random. Missing data to any question would only be excluded from that respective question instead of the whole study.

## Results

### Characteristics of study populations

A total of 1,398 respondents were recruited across the region– 702 PLHIV, 551 individuals from KPs, and 145 HCPs.

Across the region, the mean age (±SD) of PLHIV was 37.7±10.3 years, with the highest proportion of PLHIV aged 31–40 years (36.7%). The majority (89.1%) were males and 6% were females; 76.9% PLHIV were gay and 15% were bisexual (Table 1).

Respondents of KPs were younger, with a mean age (±SD) of 33.9±8.9 years, and 42.9% were aged 21–30 years. The majority (77.2%) were males and 10.2% were transwomen; 67.0% were gay and 16.8% were bisexual (Table 1)

Across the region, HCPs comprised of mainly specialists (infectious disease/HIV) (90.0%). The average (±SD) number of years of clinical practice was 12.8±7.6 years, with 29.5% having between 11–15 years of clinical practice (Table 1).

Detailed characteristics of the study populations in each country/territory are described in S1 and S2 Tables.

### Impact of COVID-19 among people living with HIV (PLHIV)

#### Access to hospitals/clinics

**Fig 1:**
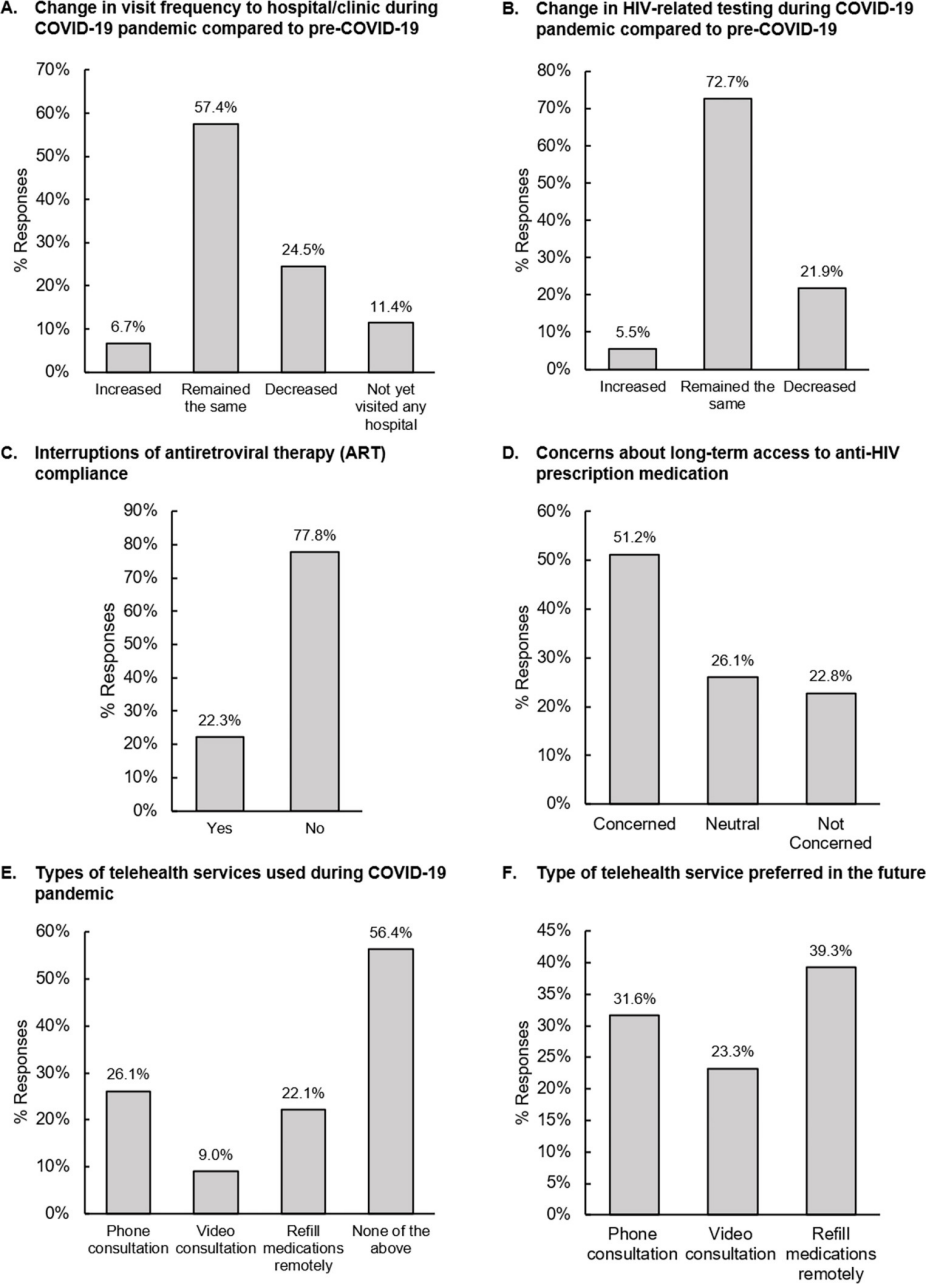
Impact of COVID-19 on HIV care and prevention perceived by people living with HIV (PLHIV) and key populations (KPs) across the region. (A) Perceived changes in visitation frequency to hospitals/clinics and (B) HIV-related testing frequency; (C) perceived interruptions by PLHIV towards antiretroviral therapy (ART) compliance; (D) perceived changes by KPs towards HIV preventive medicine (PrEP/nPEP) compliance; (E) level of concern about long-term access to anti-HIV medications such as ART or preventive medication.

Across the region, 57.4% of PLHIV reported no changes in visitation frequency to hospitals/clinics, while 24.5% reported decreased frequency of visitations and 11.4% had yet to visit any hospital/clinic during the pandemic compared to pre-COVID period (Fig 1A). Disruption in visitation frequency was more prominent in Philippines and India with 37.0% and 36.2% experiencing reduced visits to the hospital/clinic, respectively (S3 Table).

#### Access to HIV RNA viral load testing

The majority (72.7%) reported their routine viral load tests were not affected during the pandemic, while 21.9% experienced decreased frequency in HIV-related tests (Fig 1B). The PLHIV in Hong Kong and Philippines were the most affected with about 4 in 10 reporting decreasing HIV-related testing frequency (S3 Table).

Reasons perceived by PLHIV for disrupted testing frequency included travel constraint (47.4%), concerns of getting infected with COVID-19 (43.4%), and doctors recommending less frequent testing (22.7%) (Table 2).

**Table 2:** Reasons perceived for the changes in HIV testing and anti-HIV medication consumption among PLHIV and KPs as well as changes in anti-HIV medication prescription refilling among HCPs.

**Main reasons perceived by PLHIV and KPs across the region**^†^ **for the changes in HIV testing frequency**		
	**PLHIV**	**KPs**
**Base**	143	551
*Recommended less testing*	22.7%	5.6%
*Travel constraint*	47.4%	33.5%
*Fear of COVID-19 infection*	43.4%	40.3%
*Financial constraint*	19.1%	14.3%
*Reduced engagement in behaviours that increase the risks of HIV acquisition and transmission*	n/a	52.0%
*Discontinuation of free testing services at public healthcare centres/hospitals*	n/a	16.6%
*Others*	17.1%	n/a
**Main reasons perceived by PLHIV and KPs across the region**^†^ **for the changes in anti-HIV medication consumption**		
	**PLHIV**	**KPs**
**Base**	60	49
*Travel constraint*	40.6%	41.8%
*Financial constraint*, *e*.*g*., *lost job and health insurance*	28.9%	20.3%
*Doctors did not fill / refill the prescriptions*	26.9%	2.2%
*Concerns of getting infected of COVID in hospitals/clinics*	24.0%	18.0%
*As COVID-19 continues to surge in many parts of the world*, *remote access*, *and delivery of HIV services via telehealth services may become the “new normal”*.	23.2%	36.7%
*Reduced engagement in behaviours that increase the risks of HIV acquisition and transmission*	n/a	57.7%
*Cannot complete routine HIV-related testing*	n/a	21.5%
**Reasons for the changes perceived by prescribers across the region for increase or decrease of prescription medication (ART / prevention medication) refills in PLHIV and KPs**		
	**in PLHIV**	**in KPs**
**Base**	73	75
*People’s modified risky/ unsafe practices*	n/a	52.2%
*Travel constraint*	66.5%	75.0%
*Patient’s willingness / preference*	43.2%	36.6%
*Level of stock in hospitals/ clinics/ pharmacies*	9.5%	11.5%

^†^Weighted average across all 10 countries/territories (n = 145)

n/a indicate the option response is not applicable

ART, antiretroviral therapy; HCPs, healthcare providers; KPs, key populations; PLHIV, people living with HIV

#### Adherence of antiretroviral therapy during COVID-19 pandemic

Across the region, 22.3% reported interruptions in ART (Fig 1C). More than half in India (58.6%) and Thailand (54.8%) reported interruptions in ART compared to other countries/territories (S3 Table).

“Travel constraint” (40.6%), “financial constraint” (28.9%), and “doctors did not fill/refill the prescriptions” (26.9%) were among the top reasons perceived by PLHIV in the region for disruptions in their prescription refills (Table 2).

#### Concerns for future HIV care and perception of telehealth services

Overall, 51.2% of PLHIV expressed concerns towards long-term ability to access ART as the world continues to be impacted by COVID-19 (Fig 1E). More than 80% of PLHIV in Vietnam (90.6%) and the Philippines (82.7%) were concerned about long-term ART medication access (S3 Table).

**Fig 2:**
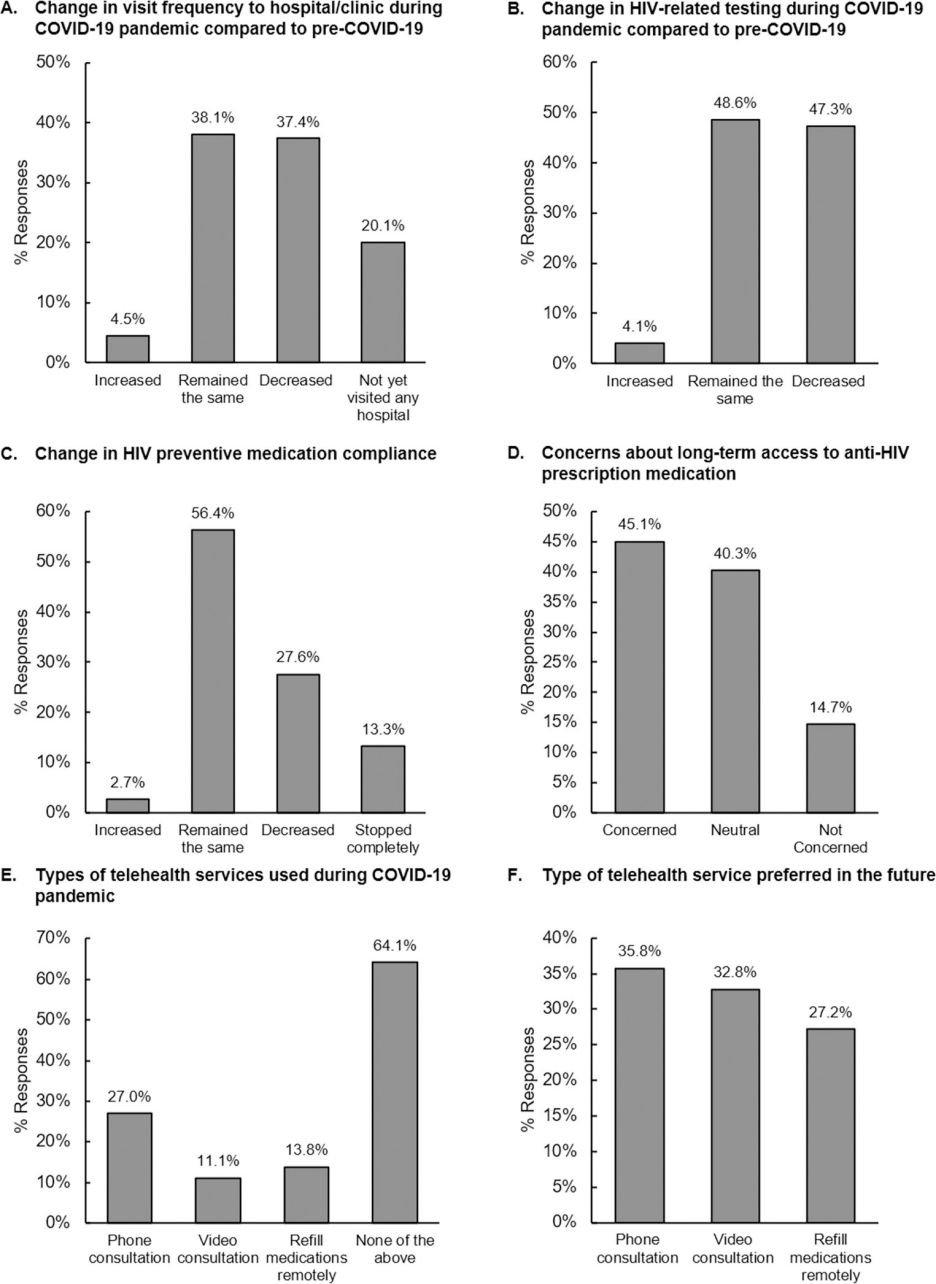
Use of telehealth services during COVID-19 by people living with HIV (PLHIV) and key populations (KPs) across the region. (A) Types of telehealth services used during COVID-19 pandemic, and (B) types of telehealth services for future HIV care and prevention preferred by PLHIV and individuals from KPs.

During the pandemic, the proportions of PLHIV utilising telehealth services such as phone consultation, video consultation, and remote refill of medications were 26.1%, 9.0% and 22.1%, respectively (Fig 2A). On the other hand, 56.4% of PLHIV across the region (Japan: 80.0%; Korea: 86.6%) did not adopt any of the above-mentioned telehealth services (S3 Table). About one-third expressed their preferences towards refilling of medications remotely (39.3%) or phone consultation with doctors/counsellors (31.6%) for future HIV telehealth services (Fig 2B).

### Impact of COVID-19 among Key Populations (KPs)

#### Access to hospital/clinics

Among KPs, 37.4% reported reduced hospital/clinic visitation frequency and 20.1% had yet to visit any hospital/clinic during the COVID-19 pandemic (Fig 1A). Individuals from KPs in Singapore (55.6%), Japan (47.8%) and Philippines (46.3%) were more affected by the pandemic than other countries/territories in terms of reduced hospital/clinic visitation frequency (S4 Table).

#### Access to HIV testing

Decreased HIV testing across the region was reported by 47.3% of KPs (Fig 1B), with Singapore being the most affected (88.9%), followed by Philippines (65.9%) (S4 Table).

Top reasons for changes in the HIV testing cited by KPs across the region included “reduced engagements in behaviours that increase risks of HIV acquisition and transmission” (52.0%), “concerns of getting infected with COVID-19” (40.3%), and “travel constraint” (33.5%) (Table 2).

#### Adherence of HIV preventive medication during COVID-19

About one-quarter (158/551) of KPs were prescribed HIV preventive medication (PrEP/nPEP); among them, 40.9% reported decreased or a complete stop in taking PrEP/nPEP (Fig 1D). Reduced consumption or complete stop of PrEP/nPEP was reported in more than 50% of KPs in Korea, Philippines, and Singapore. On the other hand, more than 70% of KPs in Hong Kong, Taiwan, and Thailand reported no changes in their PrEP/nPEP consumption (S4 Table).

Reasons cited for the perceived changes in PrEP/nPEP consumption included “reduced engagements in behaviours which increase risks of HIV acquisition and transmission” (57.7%), “travel constraint” (41.8%) and “reduced frequency of hospital/clinic visits” (36.7%) (Table 2).

#### Concerns for future HIV care and perception of telehealth services

Across the region, 45.1% taking PrEP/nPEP were concerned about the long-term ability to access preventive medications (Fig 1E).

Phone consultation, video consultation and remote refilling of medications were adopted by 27.0%, 11.1% and 13.8%, respectively. About 64.1% of KPs did not adopt any telehealth service (Fig 2A). More than half in India and Vietnam utilised phone consultation and one-third in Singapore utilised telehealth service to refill medications remotely (S4 Table).

“Phone consultation with doctor/counsellor” (35.8%) was the most preferred telehealth service across the region, followed by video consultation (32.8%) and remote refill of medications (27.2%) for HIV prevention to care continuum in the future (Fig 2B). Phone consultation was more preferred by KPs in Thailand and India, while video consultation was preferred in Taiwan and Philippines. Remote refill of medications was more preferred by KPs in Singapore and Japan (S4 Table).

### Impact of HIV care delivery among Healthcare Providers (HCPs)

#### Patient (PLHIV and KPs) load during COVID-19

**Fig 3:**
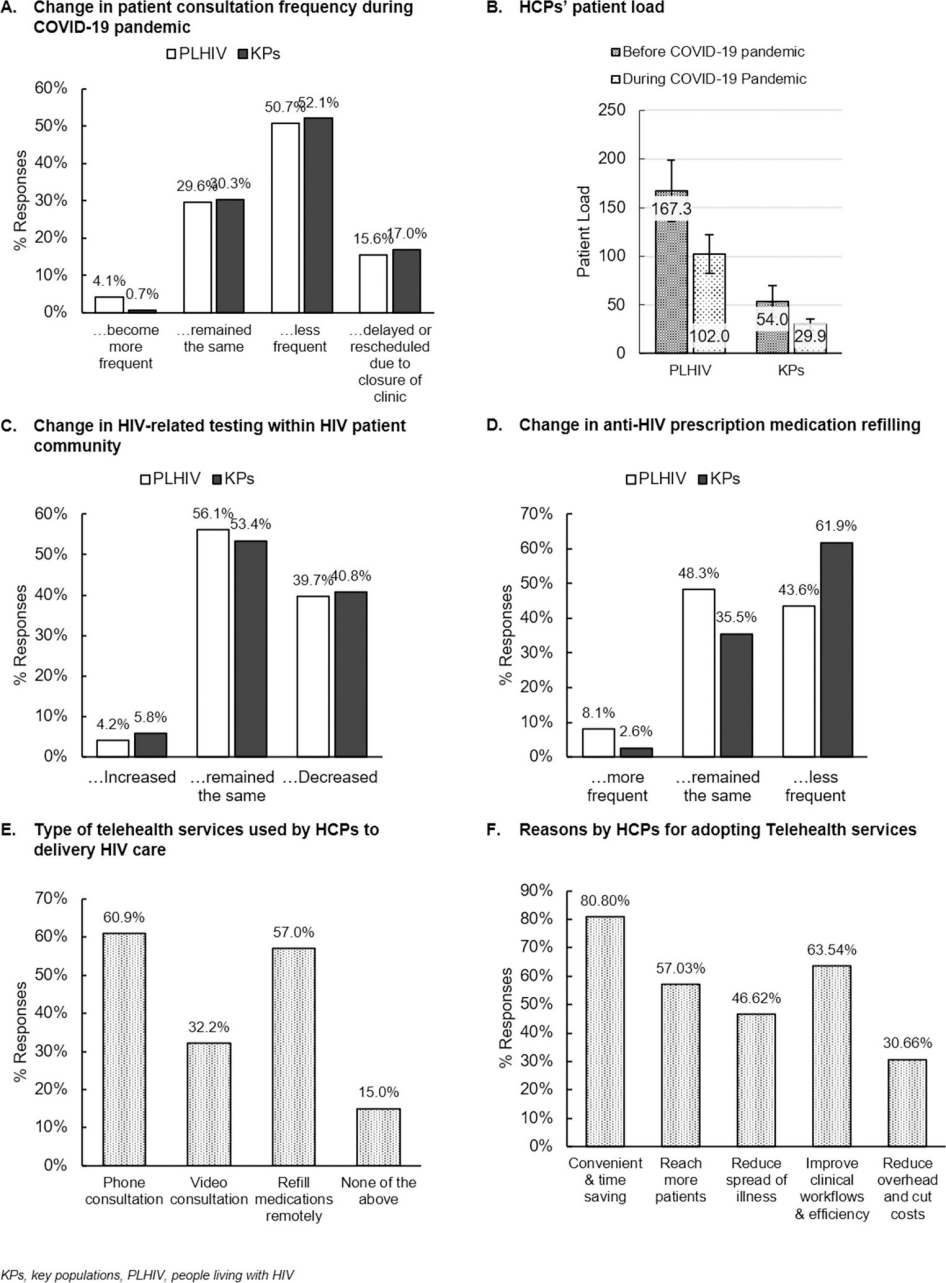
Impact of COVID-19 on HIV care among HCPs across the region. Changes in (A) consultation frequency and (B) patient load, (C) HIV-related testing frequency as well as (D) anti-HIV medication refilling frequency, (E) the types of telehealth services used, and (F) reasons for adopting telehealth services for delivering HIV prevention and care by HCPs to PLHIV and KPs.

Across the region, 50.7% and 52.1% of HCPs perceived to experience decreased consultation frequencies with PLHIV and KPs, respectively, during the pandemic. About one-tenth reported delaying or rescheduling visits of PLHIV (15.6%) or KPs (17.0%) during the pandemic due to clinic closure (Fig 3A). There was a greater decrease in patient load during the pandemic observed in KPs (44.6%: pre-COVID-19: 54.0±16.2 vs. COVID-19: 29.9±5.9, mean ± standard error) than in the PLHIV population (38.9%; 167.3±31.3 vs. 102.0±19.9) (Fig 3B). The HCPs in Philippines and Vietnam saw a greater decrease in PLHIV load than other countries/territories, while India and Vietnam reported a greater decrease of patient load in KPs (S5 Table).

#### HIV testing and/or HIV viral load monitoring

Frequency of routine HIV testing or viral load monitoring among PLHIV and KPs was reported to remain the same or increased by >60% of HCPs across the region (Fig 3C). Decreased frequency in HIV testing or viral load monitoring were reported by >70% of HCPs in India and Philippines (S5 Table).

#### Delivery of antiretroviral therapy and prevention medication refills

A higher proportion of HCPs reported reductions in refilling PrEP/nPEP for KPs (61.9%) compared to refilling ART for PLHIV (43.6%) (Fig 3D). This trend was observed across the region except for Korea and Vietnam, where more HCPs experienced reductions in refilling ART medications (Korea: 44.4%; Vietnam: 85.7%) than PrEP/nPEP (14.3%; 71.4%). The HCPs from Hong Kong, India, Philippines, Korea, Taiwan, and Vietnam reported increased frequency of prescribing medications for PLHIV and/or KPs during the pandemic (S5 Table).

Reasons perceived by HCPs for these changes in prescribing medication refills included “travel constraint” (PLHIV: 66.5%; KPs: 75.0%) and patient willingness/preferences (43.2%; 36.6%). Modified behaviours which could reduce the risk of HIV acquisition and transmission were also perceived by 52.2% of HCPs for the changes in refilling frequency observed among KPs (Table 2).

#### Adoption of telehealth services

Across the region, more than half provided remote medication refill (through community pharmacy) (57.0%) and phone consultation (60.9%), while 15.0% of HCPs did not adopt any form of telehealth service to deliver healthcare services (Fig 3E).

“Convenient and time saving” (80.8%), “improved clinical workflows and increased efficiency” (63.5%), and increased coverage of patients (57.0%) were the main drivers perceived by HCPs towards adopting telehealth services (Fig 3F).

Detailed responses of the HCPs in each country/territory are described in S5 Table.

## Discussion

This study reported that HIV prevention and care was negatively impacted by the COVID-19 pandemic and was experienced differently by PLHIV, KPs, and HCPs in different countries/territories in Asia.

The PLHIV and KPs across all ten Asian countries/territories self-reported experiencing disruptions in all aspects of HIV services, i.e., decreased hospitals/clinics visits, HIV tests, and anti-HIV medication consumption. Across the region, HCPs perceived interferences in delivery of healthcare to the HIV community, with decreased patient load, HIV-related testing frequencies, as well as anti-HIV medication refilling frequency. These observations were also reflected in Africa, China, Europe, and the US, with reports of decreased HIV viral load or routine testing/monitoring, interferences in ART refilling, adherence, and compliance, as well as accessing HIV health centres during the pandemic [12, 15–17].

Travel constraints were not only cited in this study, but also globally [9, 12, 16]. ‘Lockdown’ or shelter-in-place measures, inter-provincial/state travel restrictions and/or curfews across Asia led to residents moving away from their usual HIV care centres [18–21], interfering with their access routine HIV services [11, 12, 20, 22]. Concerns of being infected with severe acute respiratory syndrome coronavirus-2 (SARS-CoV-2) during hospital/clinic visits were also reported in other studies [10, 16, 17]. Recent studies have found that PLHIV have increased odds of severe illness or death due to SARS-CoV-2 infection [1, 23, 24]. To reduce potential exposure in health facilities, many PLHIV and KPs sought assistance from community-based organizations (CBOs) or AIDS service organizations (ASOs) to meet their HIV prevention and care needs [25].

A higher proportion of KP self-reported reduced hospital/clinic visitations, HIV-related tests, and anti-HIV medicine consumption than PLHIV. In Thailand, KPs were provided counselling for PrEP effective use, allowing the option to tailor their use according to their HIV-exposure risks [26]. This could explain for the higher proportion of Thai KPs reducing or stopping their anti-HIV medication consumption due to decreased or non-engagement of behaviours that increase the risk of HIV acquisition and transmission (e.g., condom-less anal or vaginal sex with single or multiple partners or a partner of unknown HIV serostatus, or needle sharing to inject drugs) [27, 28], thereby lessening the need for routine HIV testing and anti-HIV medication consumption. While impacted ART inventory was reported in various parts of the world [9, 29, 30], less than 10% HCPs across the region perceived the level of ART stock in the hospitals/clinics/pharmacies as a contributing factor for the changes in ART refills compared to that perceived by 26.9% of PLHIV. Reasons for these differences in perceptions warrant further investigations.

In view of travel restrictions and quarantine measures happening globally, HCPs adopted telemedicine/telehealth services to overcome barriers imposed by social and/or medical distancing to provide continuous healthcare to the community in need [31]. The HCPs prioritized convenience, improved workflows and efficiency, and wider outreach to PLHIV and KPs for continuing telehealth services to deliver HIV prevention and care services post-COVID-19 pandemic. However, more than half of PLHIV/KPs across the region did not use telehealth services, despite majority of HCPs providing remote HIV prevention and care through telemedicine. This suggests a possible gap in the delivery and access of routine HIV health services through telehealth by the HCPs and PLHIV/KPs, respectively. One of the key barriers is the lack of universal policies/guidelines for telehealth in many countries [32, 33]. For example, more respondents among Korean PLHIV/KPs preferred remote medication refilling compared to the phone consultation service used by the Korean HCPs. This suggests an unmet need within the region to understand patients’ preferences to deliver quality HIV care via telemedicine during and post-COVID-19 pandemic. Therefore, it would be essential to explore PLHIV and KP preferences and values when it comes to HIV service delivery through telehealth.

The perceived impact of COVID-19 on HIV prevention to care continuum varied across individual country/territory in Asia. PLHIV and KPs in India and Philippines appeared to experience greater HIV care access disruptions as more HCPs in both countries self-reported rescheduling/delaying patient appointments due to clinic closures. Reports of disrupted access to HIV treatment hubs due to distance and public transportation restrictions [20, 34, 35] were similarly echoed as reasons for reduced HIV tests and anti-HIV medication by Filipino PLHIV and KPs. The collaborative efforts between CBOs/ASOs and the Filipino government to collect and distribute ART refills from treatment centres to PLHIV [20, 35], could account for less PLHIV in Philippines self-reporting interruptions in ART medication consumption. However, 62.5%-82.7% still expressed concerns over their long-term access to anti-HIV medication as COVID-19 prevails. This hints at a potential gap in anti-HIV medication delivery and access locally despite efforts to overcome the barriers imposed by COVID-19 mitigation measures.

Noteworthy, fewer disruptions in HIV prevention to care continuum in Taiwan and Thailand were observed across all three study populations. Taiwan and Thailand were among countries/territories where COVID-19 local transmission rates had fallen near-zero since April 2020 [36, 37]. The relaxation of mitigation measures and return towards ‘normalcy’ of both countries earlier than the rest of the world, with necessary preventive measures in place [38, 39], could explain for the least self-reported clinic closures in Taiwan and Thailand during the pandemic. This earlier return to ‘normalcy’ could account for the lower proportion of patient community in Taiwan and Thailand being concerned about their long-term ability to access anti-HIV medication.

Notably, a moderate proportion (~60%) of the patient community in Malaysia and Korea expressed concerns towards accessing anti-HIV medication as COVID-19 prevails. The emergence of new clusters and increasing numbers of critical cases between March–October 2020 in Malaysia [40, 41] imposed a growing pressure on the nation’s public healthcare infrastructure with a prioritization for COVID-19 care [42]. The support for HIV care delivery in Malaysia was delegated through postal/courier services or multi-month dispensing of anti-HIV medication [43], potentially reducing the need for hospitals/clinics visits.

Overall, this is the first study to provide a comparative landscape of the impact of COVID-19 pandemic on the quality and accessibility of HIV services among PLHIV, KP and healthcare providers across Asia.

### Limitations

There are some limitations of this study. This is a cross-sectional and self-reported study and is subjected to recall bias. Data validation could not be performed, and no causal associations could be made. As this is an online survey, respondents without internet or comfort with online administration were potentially underrepresented in this study. As this study is explorative and descriptive in nature, factors associated with HIV care disruptions during the pandemic including respondent’s characteristics (e.g., residence type, education, underlying comorbidities, job/financial/food insecurity), HIV prevalence or geographical distribution of HIV hospital/clinic, or duration/severity of lockdown measures in each country/territory were not investigated during the study.

Furthermore, the small sample size in each study population in the countries/territories across the region might not be representative of the HCPs, PLHIV and KP populations living in the countries/territories where the study was conducted. The small sample sizes could potentially attribute to the discordant responses from patients and HCPs while simultaneously limiting the depth of understanding on the level of HIV care and prevention disruption in each country/territory. For instance, in Japan, all recruited KP respondents self-reported having not received prescriptions for anti-HIV preventive medication (e.g., PrEP/PEP). As such, this data had limited the understanding of HIV care and prevention disruption experienced by KPs in Japan to that of hospital/clinic accessibility and relevant HIV test frequency. Similarly, the absence of HCPs recruited from Malaysia further restricted the understanding of the healthcare situation in Malaysia during the pandemic. Therefore, further research on a larger sample size would be warranted to enhance the robustness of the findings.

Incidentally, with the consideration that the pandemic had led to the redirection of most healthcare resources relevant to infectious diseases to fighting the waves of SARS-CoV-2 infections nationally and globally, the observed small sample sizes during recruitment could potentially be associated with the level of disruption on HIV care and prevention in the respective countries due to the pandemic.

## Conclusion

COVID-19 disrupted access to HIV testing and continuation of anti-HIV medication in Asia. The varied impact of COVID-19 on the HIV prevention to care continuum across each country/territory could be attributed to the severity of the pandemic and different local mitigation measures implemented to address COVID-19 burden. Fewer PLHIV and KPs adopted telehealth services, albeit more HCPs delivered HIV care through telehealth. As COVID-19 continues to surge in many parts of the world, remote access, and delivery of HIV services via telehealth services may become the “new normal”. Moving forward, it will be important to continue to implement strategies that promote HIV services access in Asia including exploring PLHIV, KPs, and HCP preferences and values related to telehealth models of care.

## Supporting information

S1 Table Characteristics of respondents from people living with HIV (PLHIV) and key populations (KPs) in each country/territory. (PDF)

S2 Table Characteristics of healthcare providers (HCPs) in each country/territory. (PDF)

S3 Table Perceived changes by people living with HIV (PLHIV) towards HIV care and prevention access during COVID-19 pandemic in each country/territory—hospital/clinic visit frequency, HIV-related tests, HIV antiretroviral therapy (ART) compliance, and telemedicine services. (PDF)

S4 Table Perceived changes by individuals within the key populations (KPs) towards HIV care and prevention access during COVID-19 pandemic in each country/territory—hospital/clinic visit frequency, HIV-related tests, HIV preventive medication compliance, and telemedicine services. (PDF)

S5 Table Perceived changes by healthcare professionals (HCPs) towards HIV care and prevention delivery during COVID-19 pandemic in each country/territory–patient load, HIV-related tests, HIV preventive medication refills, and telemedicine services. (PDF)

## List of abbreviations

ART: antiretroviral therapy
ASOs: AIDS service organizations
CBOs: community-based organizations
COVID-19: coronavirus disease 2019
KPs: key populations
nPEP: non-occupational post-exposure prophylaxis
PLHIV: people living with HIV
PrEP: pre-exposure prophylaxis
