# Supplementary material for: Impact of COVID-19 on the HIV care continuum in Asia: Insights from people living with HIV, key populations, and HIV healthcare providers
Source: PLoS One. 2022 Jul 20;17(7):e0270831. doi: 10.1371/journal.pone.0270831 (PMC9299301; doi:10.1371/journal.pone.0270831)
Supplement: S1 Table — (PDF) [file pone.0270831.s001.pdf]

Table S1. Characteristics of respondents from people living with HIV (PLHIV) and key populations (KPs) in each country/territory.

	HK	IN	JP	MY	PH	SG	SK	TW	TH	VN
**PLHIV**										
**Base**	50	58	25	225	81	22	112	66	31	32
**Age (years)**										
Mean ± SD	39.2 ± 11.4	37.5 ± 9.7	50.6 ± 7.8	34.6 ± 8.1	34.1 ± 6.3	37.3 ± 10.1	38.5 ± 10.2	37.3 ± 9.3	37.2 ± 10.7	30.8 ± 6.7
21-30	26.0%	24.1%	-	32.9%	24.7%	31.8%	27.7%	24.2%	32.3%	56.3%
31-40	34.0%	44.8%	8.0%	48.0%	66.7%	27.3%	27.7%	43.9%	32.3%	34.4%
41-50	20.0%	19.0%	44.0%	15.1%	6.2%	31.8%	33.0%	21.2%	25.8%	9.4%
51-60	14.0%	10.3%	28.0%	3.1%	2.5%	9.1%	9.8%	10.6%	3.2%	-
≥61	6.0%	1.7%	20.0%	0.9%	-	-	1.8%	-	6.5%	-
**Gender**										
Male	98.0%	48.3%	100.0%	93.8%	95.1%	90.9%	100.0%	97.0%	87.1%	81.3%
Female	2.0%	32.8%	-	4.0%	-	9.1%	-	3.0%	-	9.4%
Transman	-	1.7%	-	0.4%	-	-	-	-	-	-
Transwoman	-	15.5%	-	1.3%	-	-	-	-	3.2%	6.3%
Gender-nonconforming	-	-	-	-	3.7%	-	-	-	6.5%	3.1%
Prefer not to answer	-	1.7%	-	0.4%	1.2%	-	-	-	3.2%	-
**Sexual Orientation**										
Bisexual	4.0%	51.7%	8.0%	22.7%	22.2%	9.1%	12.5%	10.6%	6.5%	3.1%
Gay	86.0%	25.9%	92.0%	64.4%	77.8%	81.8%	83.0%	83.3%	90.3%	84.4%
Lesbian	-	1.7%	-	0.9%	-	-	-	-	-	-
Straight	10.0%	12.1%	-	6.2%	-	9.1%	2.7%	6.1%	3.2%	9.4%
Other (e.g., pansexual, trans-sexual, non-sexual)	-	6.9%	-	2.2%	-	-	0.9%	-	-	-
Prefer not to answer	-	1.7%	-	3.6%	-	-	0.9%	-	-	3.1%
**KPs**										
**Base**	27	32	23	41	41	9	174	104	41	59
**Age (years)**										
Mean ± SD	38.7 ± 11.1	35.1 ± 9.2	36.6 ± 11.3	31.4 ± 7.4	35.4 ± 8.9	34.4 ± 7.8	33.5 ± 8.0	33.5 ± 7.7	32.6 ± 7.5	27.5 ± 4.5
21-30	25.9%	37.5%	39.1%	56.1%	31.7%	33.3%	42.0%	38.5%	43.9%	81.4%

31-40	37.0%	34.4%	26.1%	29.3%	43.9%	44.4%	38.5%	46.2%	43.9%	17.0%
41-50	18.5%	25.0%	21.7%	14.6%	19.5%	22.2%	17.2%	12.5%	9.8%	1.7%
51-60	14.8%	-	8.7%	-	2.4%	-	2.3%	2.9%	2.4%	-
≥61	3.7%	3.1%	4.4%	-	2.4%	-	-	-	-	-
**Gender**										
Male	74.1%	46.9%	73.9%	53.7%	85.4%	88.9%	97.7%	96.2%	63.4%	91.5%
Female	22.2%	3.1%	13.0%	2.4%	-	11.1%	-	3.9%	4.9%	1.7%
Transman	3.7%	9.4%	4.4%	2.4%	-	-	0.6%	-	2.4%	-
Transwoman	-	34.4%	4.4%	34.2%	2.4%	-	0.6%	-	19.5%	6.8%
Gender-nonconforming	-	-	-	2.4%	7.3%	-	-	-	4.9%	-
Prefer not to answer	-	6.3%	4.4%	4.9%	4.9%	-	1.2%	-	4.9%	-
**Sexual Orientation**										
Bisexual	11.1%	37.5%	17.4%	17.1%	14.6%	22.2%	15.5%	9.6%	9.8%	13.6%
Gay	59.3%	46.9%	73.9%	36.6%	80.5%	55.6%	82.8%	85.6%	70.7%	78.0%
Lesbian	3.7%	-	-	-	-	-	-	-	-	-
Straight	22.2%	3.1%	8.7%	22.0%	2.4%	11.1%	-	4.8%	14.6%	6.8%
Other (e.g., pansexual, trans-sexual, non-sexual)	-	6.3%	-	14.6%	2.4%	11.1%	1.7%	-	4.9%	-
Prefer not to answer	3.7%	6.3%	-	9.8%	-	-	-	-	-	1.7%

"-" indicate as no-responses recorded

HK, Hong Kong; IN, India; JP, Japan; MY, Malaysia; PH, Philippines; SG, Singapore; SK, South Korea; TW, Taiwan; TH, Thailand; VN, Vietnam
