# Supplementary material for: Impact of COVID-19 on the HIV care continuum in Asia: Insights from people living with HIV, key populations, and HIV healthcare providers
Source: PLoS One. 2022 Jul 20;17(7):e0270831. doi: 10.1371/journal.pone.0270831 (PMC9299301; doi:10.1371/journal.pone.0270831)
Supplement: S2 Table — (PDF) [file pone.0270831.s002.pdf]

Table S2. Characteristics of healthcare providers (HCPs) in each country/territory.

	HK	IN	JP	PH	SG	SK	TW	TH	VN
**Base**	7	34	6	33	6	9	32	11	7
**Specialty**									
Infectious Disease/HIV Specialist	100.0%	94.1%	100.0%	78.8%	100.0%	100.0%	96.9%	54.6%	85.7%
General Practitioner	-	5.9%	-	21.2%	-	-	3.1%	45.5%	14.3%
**Type of Hospital/Clinic§**									
Public/ Government / Restructured hospital	85.7%	17.7%	33.3%	27.3%	66.7%	22.2%	n/a	63.6%	28.6%
Private hospital	-	38.2%	16.7%	51.5%	33.3%	77.8%	n/a	9.1%	-
Private clinic	14.3%	32.4%	16.7%	9.1%	-	-	n/a	-	71.4%
Medical centre	n/a	n/a	n/a	n/a	n/a	n/a	65.6%	n/a	n/a
Regional hospital	n/a	n/a	n/a	n/a	n/a	n/a	28.1%	n/a	n/a
Area hospital	n/a	n/a	n/a	n/a	n/a	n/a	6.3%	n/a	n/a
Other	-	11.8%	33.3%	12.1%	-	-	n/a	27.3%	-
**Years of Practice**									
Mean ± SD	11.7 ± 4.9	19.5 ± 9.4	20.5 ± 6.3	10.2 ± 6.2	7.3 ± 4.5	10.1 ± 5.8	14.2 ± 7.1	13.6 ± 8.6	8.3 ± 3.7
≤ 5 years	14.3%	2.9%	-	33.3%	33.3%	33.3%	12.5%	27.3%	28.6%
6 - 10 years	14.3%	17.6%	-	21.2%	50.0%	11.1%	21.9%	9.1%	42.9%
11 - 15 years	57.1%	26.5%	33.3%	24.2%	16.7%	33.3%	28.1%	18.2%	28.6%
15 - 20 years	14.3%	14.7%	16.7%	15.2%	-	22.2%	15.6%	27.3%	-
21 - 25 years	-	5.9%	33.3%	6.1%	-	-	18.8%	9.1%	-
> 25 years	-	32.4%	16.7%	-	-	-	3.1%	9.1%	-

"-" indicate as no-responses recorded

n/a indicate response not applicable for HCPs residing outside of TW.

HK, Hong Kong; IN, India; JP, Japan; MY, Malaysia; PH, Philippines; SG, Singapore; SK, South Korea; TW, Taiwan; TH, Thailand; VN, Vietnam
