# Supplementary material for: Impact of COVID-19 on the HIV care continuum in Asia: Insights from people living with HIV, key populations, and HIV healthcare providers
Source: PLoS One. 2022 Jul 20;17(7):e0270831. doi: 10.1371/journal.pone.0270831 (PMC9299301; doi:10.1371/journal.pone.0270831)
Supplement: S3 Table — (PDF) [file pone.0270831.s003.pdf]

Table S3. Perceived changes by people living with HIV (PLHIV) towards HIV care and prevention access during COVID-19 pandemic in each country/territory - hospital/clinic visit frequency, HIV-related tests, HIV antiretroviral therapy (ART) compliance, and telemedicine services.

	PLHIV (%)									
	**HK‡**	**IN‡**	**JP**	**MY‡**	**PH‡**	**SG**	**SK‡**	**TW‡**	**TH‡**	**VN‡**
**Access to Hospital/clinics and routine HIV-tests**										
**Base**	50	58	25	225	81	22	112	66	31	32
During COVID-19, how has your visit frequency to hospital/clinic changed compared with the pre-COVID period?										
Increased	2.0%	15.5%	4.0%	4.9%	2.5%	13.6%	2.7%	3.0%	9.7%	9.4%
Remained the same	48.0%	29.3%	76.0%	70.2%	25.9%	50.0%	80.4%	83.3%	58.1%	53.1%
Decreased	28.0%	36.2%	20.0%	21.3%	37.0%	27.3%	13.4%	10.6%	19.4%	31.3%
Not yet visited any hospital	22.0%	19.0%	-	3.6%	34.6%	9.1%	3.6%	3.0%	12.9%	6.3%
During COVID-19, how has your frequency of HIV-related tests such as HIV RNA load changed compared with the pre-COVID period?										
Increased	-	25.9%	4.0%	4.9%	4.9%	4.6%	2.7%	1.5%	3.2%	3.1%
Remained the same	60.0%	53.5%	88.0%	76.0%	53.1%	72.7%	91.1%	95.5%	80.7%	56.3%
Decreased	40.0%	20.7%	8.0%	19.1%	42.0%	22.7%	6.3%	3.0%	16.1%	40.6%
**Access to HIV antiretroviral therapy (ART) among PLHIV with prescriptions**										
**Base**	50	58	25	225	81	22	112	66	31	32
During COVID-19, have you ever interrupted your ART, i.e. stopped taking the treatment or did not follow the recommended dosing?										
Yes	10.0%	58.6%	8.0%	10.2%	8.6%	31.8%	12.5%	9.1%	54.8%	18.8%
No	90.0%	41.4%	92.0%	89.8%	91.4%	68.2%	87.5%	90.9%	45.2%	81.3%
How has the frequency of taking your ART changed compared with the pre-COVID period?										
**Base**	5	34	2	23	7	7	14	6	17	6
Decreased	20.0%	50.0%	-	34.8%	28.6%	71.4%	42.9%	50.0%	41.2%	33.3%
Stopped completely	20.0%	11.8%	-	-	-	28.6%	-	-	5.9%	16.7%
Others (e.g., based on doctor's advice, forgot some few doses)	60.0%	38.2%	100.0%	65.2%	71.4%	-	57.1%	50.0%	52.9%	50.0%
Looking forward in a world that continues to be impacted of COVID-19, how concerned are you about your longer-term ability to access HIV ART?										
Concerned	40.0%	50.0%	20.0%	62.2%	82.7%	72.7%	35.7%	28.8%	29.0%	90.6%
Neutral	40.0%	41.4%	12.0%	25.3%	8.6%	9.1%	27.7%	51.5%	35.5%	9.4%
Not Concerned	20.0%	8.6%	68.0%	12.4%	8.6%	18.2%	36.6%	19.7%	35.5%	-

**Usage of Telemedicine services**										
**Base**	50	58	25	225	81	22	112	66	31	32
What types of telemedicine services, e.g. receiving healthcare services remotely via the use of technology, e.g. mobile phone, tablet, computer, have you received from your doctor during COVID-19?										
Provided a phone consultation	10.0%	65.5%	16.0%	32.0%	37.0%	22.7%	10.7%	3.0%	45.2%	18.8%
Provided a video consultation	16.0%	10.3%	-	8.9%	37.0%	4.6%	0.9%	3.0%	3.2%	6.3%
Provided to refill medications remotely	30.0%	22.4%	4.0%	42.2%	64.2%	9.1%	1.8%	28.8%	6.5%	12.5%
None of the above	62.0%	17.2%	80.0%	39.1%	17.3%	68.2%	86.6%	69.7%	51.6%	71.9%
What is your most preferred telemedicine service in the future?										
Phone consultation with doctor/counsellor	22.0%	55.2%	24.0%	31.1%	12.4%	13.6%	57.1%	24.2%	48.4%	28.1%
Video consultation with doctor/counsellor	22.0%	15.5%	36.0%	24.4%	38.3%	36.4%	13.4%	27.3%	16.1%	3.1%
Refill medications remotely	52.0%	27.6%	32.0%	39.1%	44.4%	36.4%	24.1%	45.5%	29.0%	62.5%
Other	4.0%	1.7%	8.0%	5.3%	4.9%	13.6%	5.4%	3.0%	6.5%	6.3%

‡*Sample size of at least 30 is considered robust*

"-" indicate as no-responses recorded

HK, Hong Kong; IN, India; JP, Japan; MY, Malaysia; PH, Philippines; SG, Singapore; SK, South Korea; TW, Taiwan; TH, Thailand; VN, Vietnam
