# Supplementary material for: Impact of COVID-19 on the HIV care continuum in Asia: Insights from people living with HIV, key populations, and HIV healthcare providers
Source: PLoS One. 2022 Jul 20;17(7):e0270831. doi: 10.1371/journal.pone.0270831 (PMC9299301; doi:10.1371/journal.pone.0270831)
Supplement: S4 Table — (PDF) [file pone.0270831.s004.pdf]

Table S4. Perceived changes by individuals within the key populations (KPs) towards HIV care and prevention access during COVID-19 pandemic in each country/territory - hospital/clinic visit frequency, HIV-related tests, HIV preventive medication compliance, and telemedicine services.

	KP (%)									
	**HK‡**	**IN‡**	**JP§**	**MY‡**	**PH‡**	**SG**	**SK‡**	**TW‡**	**TH‡**	**VN‡**
**Access to Hospital/clinics and routine HIV-tests**										
**Base**	27	32	23	41	41	9	174	104	41	59
During COVID-19, how has your visit frequency to hospital/clinic changed compared with the pre-COVID period?										
Increased	3.7%	6.3%	-	7.3%	-	-	6.3%	7.7%	12.2%	1.7%
Remained the same	37.0%	25.0%	34.8%	41.5%	19.5%	33.3%	34.5%	62.5%	58.5%	33.9%
Decreased	37.0%	31.3%	47.8%	29.3%	46.3%	55.6%	40.8%	27.9%	17.1%	40.7%
Not yet visited any hospital	22.2%	37.5%	17.4%	22.0%	34.2%	11.1%	18.4%	1.9%	12.2%	23.7%
During COVID-19, how has your frequency of HIV testing changed compared with the pre-COVID period?										
Increased	-	6.3%	4.4%	-	2.4%	11.1%	4.0%	2.9%	4.9%	5.1%
Remained the same	59.3%	43.8%	39.1%	68.3%	31.7%	-	45.4%	73.1%	70.7%	54.2%
Decreased	40.7%	50.0%	56.5%	31.7%	65.9%	88.9%	50.6%	24.0%	24.4%	40.7%
**Access to HIV preventive medicine among KPs with prescriptions**										
**Base**	5	9	0	10	16	4	8	38	26	42
During COVID-19, how has the frequency of taking your HIV preventive medicine changed compared with the pre-COVID period?										
Increased	-	-	n/a	-	-	-	-	13.2%	3.9%	2.4%
Remained the same	80.0%	55.6%	n/a	60.0%	43.8%	-	50.0%	76.3%	73.1%	66.7%
Decreased	20.0%	22.2%	n/a	40.0%	31.3%	75.0%	25.0%	10.5%	11.5%	19.1%
Stopped completely	-	22.2%	n/a	-	25.0%	25.0%	25.0%	-	11.5%	11.9%
Looking forward in a world that continues to be impacted of COVID-19, how concerned are you about your longer-term ability to access HIV preventive medicine?										
Concerned	40.0%	44.4%	n/a	70.0%	62.5%	50.0%	62.5%	29.0%	30.8%	45.2%
Neutral	60.0%	33.3%	n/a	30.0%	25.0%	50.0%	12.5%	57.9%	38.5%	38.1%
Not Concerned	-	22.2%	n/a	-	12.5%	-	25.0%	13.2%	30.8%	16.7%
**Usage of Telemedicine services**										
**Base**	27	32	23	41	41	9	174	104	41	59

What types of telemedicine services, e.g., receiving healthcare services remotely via the use of technology, e.g. mobile phone, tablet, computer, have you received from your doctor during COVID-19?										
Provided a phone consultation	29.6%	59.4%	-	36.6%	14.6%	33.3%	5.2%	1.9%	31.7%	57.6%
Provided a video consultation	11.1%	18.8%	-	17.1%	29.3%	11.1%	-	4.8%	4.9%	13.6%
Provided to refill medications remotely	18.5%	6.3%	-	19.5%	14.6%	33.3%	0.6%	14.4%	7.3%	23.7%
None of the above	66.7%	34.4%	100.0%	56.1%	58.5%	55.6%	94.3%	82.7%	61.0%	32.2%
What is your most preferred telemedicine service in the future?										
Phone consultation with doctor/counsellor	44.4%	50.0%	21.7%	36.6%	26.8%	11.1%	35.1%	25.0%	61.0%	45.8%
Video consultation with doctor/counsellor	37.0%	31.3%	30.4%	34.2%	61.0%	22.2%	23.6%	51.0%	17.1%	20.3%
Refill medications remotely	14.8%	9.4%	47.8%	26.8%	7.3%	55.6%	37.9%	24.0%	19.5%	28.8%
Other	3.7%	9.4%	-	2.4%	4.9%	11.1%	3.5%	-	2.4%	5.1%

‡Sample size of at least 30 is considered robust

§Japanese respondents from KPs has self-reported not being prescribed with HIV preventive medicine

"-" indicate as no-responses recorded

n/a indicate a response was not applicable

HK, Hong Kong; IN, India; JP, Japan; MY, Malaysia; PH, Philippines; SG, Singapore; SK, South Korea; TW, Taiwan; TH, Thailand; VN, Vietnam
