# Supplementary material for: Impact of COVID-19 on the HIV care continuum in Asia: Insights from people living with HIV, key populations, and HIV healthcare providers
Source: PLoS One. 2022 Jul 20;17(7):e0270831. doi: 10.1371/journal.pone.0270831 (PMC9299301; doi:10.1371/journal.pone.0270831)
Supplement: S5 Table — (PDF) [file pone.0270831.s005.pdf]

Table S5. Perceived changes by healthcare professionals (HCPs) towards HIV care and prevention delivery during COVID-19 pandemic in each country/territory – patient load, HIV-related tests, HIV preventive medication refills, and telemedicine services.

	HCPs																	
	**HK‡**		**IN‡**		**JP**		**PH‡**		**SG‡**		**SK‡**		**TW‡**		**TH**		**VN‡**	
**Patient Load and routing tests**																		
**Base**	7		34		6		33		6		9		32		11		7	
During COVID-19, how do you feel the interval/frequency of visit for "PLHIV/ KPs" has changed compared with the pre-COVID-19 period?																		
**Affected patient population:**	**PLHIV**	**KPs**	**PLHIV**	**KPs**	**PLHIV**	**KPs**	**PLHIV**	**KPs**	**PLHIV**	**KPs**	**PLHIV**	**KPs**	**PLHIV**	**KPs**	**PLHIV**	**KPs**	**PLHIV**	**KPs**
…become more frequent	-	-	2.9%	5.9%	16.7%	-	3.0%	-	-	-	-	-	-	-	-	-	14.3%	-
…remained the same	28.6%	28.6%	14.7%	8.8%	50.0%	50.0%	-	6.1%	16.7%	33.3%	55.6%	22.2%	68.8%	40.6%	18.2%	54.6%	14.3%	28.6%
…less frequent	57.1%	57.1%	58.8%	64.7%	33.3%	33.3%	57.6%	51.5%	50.0%	33.3%	44.4%	55.6%	25.0%	56.3%	72.7%	45.5%	57.1%	71.4%
…delayed or rescheduled due to closure of clinic	14.3%	14.3%	23.5%	20.6%	-	16.7%	39.4%	42.4%	33.3%	33.3%	-	22.2%	6.3%	3.1%	9.1%	-	14.3%	-
Average patient load in a typical month (mean ± SE)																		
Before COVID-19 pandemic	65.7 ± 19.3	10.0 ± 1.9	431.7 ± 131.9	165.0 ± 83.0	129.0 ± 31.7	50.0 ± 0	93.0 ± 29.9	26.2 ± 8.2	18.3 ± 5.9	8.3 ± 3.9	147.8 ± 45.5	17.6 ± 6.4	178.8 ± 26.2	19.7 ± 3.3	135.8 ± 41.3	37.7 ± 15.9	305.3 ± 282.5	129.6 ± 48.8
During COVID-19 Pandemic	53.7 ± 18.9	8.6 ± 1.0	239.4 ± 109.5	57.9 ± 22.4	120.3 ± 26.0	30.0 ± 0	24.3 ± 7.4	22.9 ± 8.7	15.8 ± 3.7	8.3 ± 3.9	86.2 ± 20.4	16.6 ± 6.9	183.3 ± 26.5	16.3 ± 3.3	106.6 ± 34.1	27.6 ± 8.7	85.4 ± 69.4	66.6 ± 40.8
During COVID-19, how do you feel "PLHIV/KPs" access to routine HIV test/HIV RNA load test has changed compared with the pre-COVID period?																		
…Increased	-	-	8.8%	2.9%	-	-	-	3.0%	-	-	-	-	-	3.1%	-	-	28.6%	42.9%
…remained the same	100.0%	71.4%	17.7%	11.8%	83.3%	100.0%	21.2%	18.2%	100.0%	100.0%	77.8%	77.8%	68.8%	56.3%	68.8%	45.5%	-	-
…Decreased	-	28.6%	73.5%	85.3%	16.7%	-	78.8%	78.8%	-	-	22.2%	22.2%	31.3%	40.6%	31.3%	54.6%	71.4%	57.1%
**Refill of anti-HIV medication - Antiretroviral therapy (ART) for PLHIV or HIV preventive medication (PrEP/PEP) for KPs**																		
**Base**	7	5	34	25	6	n/a	33	27	6	4	9	7	32	31	11	10	7	7
What impact has COVID-19 had on the frequency of patient's refilling prescriptions (ART / prevention medications) compared to the pre-COVID period?																		
…more frequent	28.6%	-	17.7%	-	-	n/a	3.0%	-	-	-	11.1%	-	12.5%	3.2%	-	-	-	14.3%
…remained the same	57.1%	20.0%	32.4%	20.0%	50.0%	n/a	66.7%	33.3%	83.3%	25.0%	44.4%	85.7%	59.4%	45.2%	27.3%	40.0%	14.3%	14.3%
…less frequent	14.3%	80.0%	50.0%	80.0%	50.0%	n/a	30.3%	66.7%	16.7%	75.0%	44.4%	14.3%	28.1%	51.6%	72.7%	60.0%	85.7%	71.4%
**Usage of Telemedicine services**																		
**Base**	7		34		6		33		6		9		32		11		7	

Types of telehealth/ telemedicine practice adopted during COVID-19, i.e. the use of telecommunication techniques to deliver health care services									
Provided a phone consultation	14.3%	85.3%	100.0%	66.7%	66.7%	77.8%	6.3%	45.5%	85.7%
Provided a video consultation	-	50.0%	-	66.7%	50.0%	-	34.4%	45.5%	42.9%
Provided to refill medications remotely (community pharmacy)	42.9%	67.7%	16.7%	69.7%	100.0%	33.3%	43.8%	81.8%	57.1%
None of the above	57.1%	2.9%	-	6.1%	-	22.2%	37.5%	9.1%	-
Looking forward in a world that continues to be impacted of COVID-19, how do you anticipate the adoption of telehealth services offered to your patients will change??									
Increase	100.0%	76.5%	50.0%	81.8%	83.3%	55.6%	71.9%	72.7%	71.4%
Remain the same	-	17.7%	50.0%	15.2%	16.7%	44.4%	25.0%	18.2%	14.3%
Decrease	-	5.9%	-	3.0%	-	-	3.1%	9.1%	14.3%
What do you think are the main drivers in adoption of telehealth services?									
**Base**	7	26	3	27	5	5	23	8	5
Convenient & time saving	85.7%	100.0%	33.3%	92.6%	100.0%	40.0%	56.5%	87.5%	100.0%
Reach more patients	28.6%	80.8%	33.3%	85.2%	20.0%	60.0%	60.9%	50.0%	100.0%
Reduce spread of illness	14.3%	73.1%	66.7%	70.4%	-	20.0%	47.8%	62.5%	80.0%
Improve clinical workflows and increase efficiency	85.7%	50.0%	33.3%	59.3%	60.0%	60.0%	82.6%	25.0%	100.0%
Reduce overhead and cut costs	-	53.9%	33.3%	44.4%	-	-	21.7%	50.0%	80.0%

‡Note: Data considered robust for Hong Kong, India, Philippines, Singapore, and Taiwan.
India, Taiwan, and Philippines have at least 30 which was considered minimum sample size for robustness.
Data for Singapore and Hong Kong can be considered robust given the small country/territory population of relevant HCPs, minimum criteria of 5 HCPs.
n/a – not applicable as no KP respondent from Japan had self-reported to receive HIV preventive medication prescriptions
"-" indicate as no-responses recorded
ART, antiretroviral therapy; KPs, key populations; PLHIV, people living with HIV; SE, standard error
HK, Hong Kong; IN, India; JP, Japan; MY, Malaysia; PH, Philippines; SG, Singapore; SK, South Korea; TW, Taiwan; TH, Thailand; VN, Vietnam
